# Emerging Role of PD-1/PD-L1 Inhibitors in Chronic Liver Diseases

**DOI:** 10.3389/fphar.2021.790963

**Published:** 2021-12-23

**Authors:** Vishakha Singh, Amit Khurana, Prince Allawadhi, Anil Kumar Banothu, Kala Kumar Bharani, Ralf Weiskirchen

**Affiliations:** ^1^ Department of Biosciences and Bioengineering, Indian Institute of Technology (IIT) Roorkee, Roorkee, India; ^2^ Institute of Molecular Pathobiochemistry, Experimental Gene Therapy and Clinical Chemistry (IFMPEGKC), RWTH Aachen University Hospital, Aachen, Germany; ^3^ Centre for Biomedical Engineering (CBME), Indian Institute of Technology (IIT) Delhi, New Delhi, India; ^4^ Department of Veterinary Pharmacology and Toxicology, College of Veterinary Science (CVSc), Hyderabad, India; ^5^ Department of Veterinary Pharmacology and Toxicology, College of Veterinary Science (CVSc), Warangal, India

**Keywords:** programmed cell death protein 1, chronic liver diseases, hepatocellular carcinoma, T-cells, immunotherapy, monoclonal antibodies

## Abstract

Programmed cell death protein 1 (PD-1)/PD-ligand (L)1, the immune checkpoint inhibitors have emerged as a promising strategy for the treatment of various diseases including chronic liver diseases (CLDs) such as hepatitis, liver injury and hepatocellular carcinoma (HCC). The role of PD-1/PD-L1 has been widely inspected in the treatment of viral hepatitis and HCC. PD-1 is known to play a crucial role in inhibiting immunological responses and stimulates self-tolerance by regulating the T-cell activity. Further, it promotes apoptosis of antigen-specific T-cells while preventing apoptosis of T_reg_ cells. PD-L1 is a *trans*-membrane protein which is recognized as a co-inhibitory factor of immunological responses. Both, PD-1 and PD-L1 function together to downregulate the proliferation of PD-1 positive cells, suppress the expression of cytokines and stimulate apoptosis. Owing to the importance of PD-1/PD-L1 signaling, this review aims to summarize the potential of PD-1/PD-L1 inhibitors in CLDs along with toxicities associated with them. We have enlisted some of the important roles of PD-1/PD-L1 in CLDs, the clinically approved products and the pipelines of drugs under clinical evaluation.

## 1 Introduction

Programmed cell death protein 1 (PD-1) is an immune checkpoint molecule whose function is to reduce the T-cell activity for preventing autoimmune damage during immune responses. In case of chronic infections, prolonged antigen exposure results in permanent expression of PD-1 which can limit immune-mediated clearance of pathogens (Ishida et al., 1992). It is mainly an immune checkpoint inhibitory receptor expressed on immune cells which are involved in activating immunosuppressive signaling cascade. PD-1 functions by binding to its ligands namely; PD-L1 and PD-L2 and thereby prevents stimulatory signals from T-cell receptors (TCR) (Butte et al., 2008). This immune suppressive PD-1 works as brakes for regulating the acquired immunity. PD-1 is present in T-cells, B-cells, antigen presenting cells (APCs) and in few other non-lymphoid tissues. The association of ligands with PD-1 on the T-cell promote immune suppression (Butte et al., 2007). Moreover, PD-1 expression is also seen in pancreatic islets, cardiac endothelium and placenta depicting its possible functioning in immunological tolerance. PD-1 is present in its monomeric form as a surface glycoprotein and can also be placed on TCR signalosome when TCR binds with MHC complexes (Wu et al., 2009). The inducible expression of PD-1 can be seen in T-cells (CD4^+^ and CD8^+^), B-cells, macrophages, natural killer cells (NK), dendritic cells (DCs). The PD-1 expression can be stimulated on T-cells by T-cell receptor signaling and cytokines like IL-2, IL-7 and some of the interferons. The PD-1 expression is remarkably enhanced on stimulated T-cells and within 24 h, the expression can be increased depending upon the concentration of mentioned stimuli (Calderaro et al., 2016). Hence, PD-1 can also function as a marker of active T-cell. There is difference in kinetics of expression pattern of PD-1 in acute and chronic infections. For instance, in case of acute infections, the expression of PD-1 is transitory while a sustained expression is seen in cases of chronic infection (Watanabe et al., 2010). This prolonged expression in chronic infection can head up to T-cell dysfunction and make T-cells exhausted (Zhou et al., 2019). The immunosuppressive potential of PD-1 is regulated mainly by involvement with its ligands namely PD-L1 and PD-L2. The PD-L2 expression is inducible on DCs, mast cells and macrophages, while PD-L1 is constitutively expressed on B-cells, T-cells, macrophages, DCs, mast cells and mesenchymal stem cells (MSCs) (Mataki et al., 2007). Moreover, PD-1 is also seen on several non-hematopoietic cells like epithelial cells, hepatocytes, myocytes, pancreatic islet cells, vascular endothelial cells and astrocytes. Studies have shown that PD-L1 and PD-L2 mediated expression of PD-1 are independent of each other in regulating T-cell response (Iwai et al., 2003). PD-L2 is mainly known to regulate proliferation of CD8^+^T-cells while PD-L1 generally regulates peripheral T_reg_ cells. PD-1 regulates and stimulates T-cell activity by various mechanisms (Iwai et al., 2003). The involvement of PD-1 with PD-1 ligands leads to prevention of T-cell signaling and related downstream responses. When PD-1 ligands bind with PD-1, the phosphorylation of tyrosine of the cytoplasmic tail of PD-1 takes place which is followed by placement of Src homology region 2 (SH2)-containing protein tyrosine phosphatase 2 (SHP-2), which is a protein tyrosine phosphatase (PTP) (Riella et al., 2012). This functions by dephosphorylating kinases and in turn leads to inhibition of downstream signaling leading to the activation of T-cell receptors and CD28. Moreover, SHP2 can also inhibit T-cell receptor signaling by dephosphorylating the Zap70/CD3ε signalosome. The PD-1 signaling leading to immunosuppression include inhibition of extracellular-signal regulated kinase (ERK), phosphoinositide 3-kinase (PI3K), AKT, phospholipase C-γ (PLCγ) and control cell cycle resulting into reduced IFN-γ/IL-2 generation, decreased proliferative ability and enhanced apoptosis (Schönrich and Raftery, 2019). Moreover, PD-1 signaling modulates T-cell functioning by preventing glycolysis and favors lipid degradation and β-oxidation (Li et al., 2020).

Inhibitory signaling by PD-1 ligands regulates and maintains the induction and tolerance to self-antigen via PD-1 pathway. One of the mechanisms opted by PD-1 to control autoreactivity is by inducing the T_reg_ cells in peripheral circulation (Jubel et al., 2020). It is in disparity to natural T_reg_ cells, that are originated from thymic selection and express transcriptional regulator of the T_reg_ cell phenotype namely forkhead box P3 (FoxP3) (Han et al., 2020). FoxP3 is expressed by naive CD4^+^T-cells by expressing the T-cell receptor and PD-1 on its surface. Both, the induced and natural T_reg_ are known to downregulate the immune response by generating various immunomodulatory molecules namely anti-inflammatory cytokines, IL-10 and transforming growth factor-β (TGF-β) (Zak et al., 2015). PD-1 is mainly seen in activated T_reg_ cells which in turn regulates the T_reg_ cell activity. Additionally, the blocking of PD-1 results in reduction in suppression of T_reg_ cells *in vivo* (Dong et al., 1999). The activation of peripheral T_reg_ cells leads to decline the stimulation and effector activity of self-reactive CD4^+^ and CD8^+^T-cells (Yamazaki et al., 2002). Together, these data highlight the complexity of PD-1 association and the distinctive roles of PD-1 pathway in relation to infection and autoimmunity. Thus, it has been postulated that PD-1 signaling mainly works to decrease the functioning of self-reactive T-cells and also enhances the activation and effector activity of antigen-specific T-cells. Conclusively, PD-1 is crucial in regulating autoimmunity and infection as PD-1 signaling via T cells restricts immune-mediated tissue damage during infections (Selenko-Gebauer et al., 2003; Jubel et al., 2020). This review summarizes the physiological and functional role of PD-1 and its importance in CLDs.

## 2 PD-1/PD-L1 Physiology and Functional Relevance

### 2.1 PD-1

The receptor PD-1 is a protein having 288 amino acids with a N-terminal IgV-like domain. It is also known as CD279 and was first observed in IL-3 lacking LyD9 (murine hematopoietic progenitor) and 2B4-11 (murine T-cell hybridoma) cell lines (Ishida et al., 1992). It shares 13% sequence similarity with induced T-cell co-stimulator, 15% homology with CD28 and 20% similarity with CTLA4. The constitutive expression of PD-1 is present in immature thymocytes, activated CD4^+^and CD8^+^T-cells, B-cells, DCs, NK cells (Carreno and Collins, 2002). The expression of PD-1 is induced on APCs, monocytes and DCs via transcription factors namely NOTCH, Forkhead box protein (FOXO1), interferon regulatory factor (IRFs) and nuclear factor of activated T-cells (NFAT) (Ahmadzadeh et al., 2009). Additionally, IL-10 and TGF-β could stimulate the PD-1 expression in chronic infections. The increased expression of PD-1 is a marked feature of exhausted T-cells (Staron et al., 2014).

For the expression of PD-1 gene, the conserved upstream regulatory regions namely CR-B and COR-C are crucial. In the CR-C region, there is a binding site which is connected to the NFATc1 (NFAT2) in TCD4 and TCD8 units (Li et al., 2015). Additionally, in the CR-B region, c-FOS is associated leading to increased expression of PD-1. When NFATc is stimulated, it binds to the pdcd1promoter leading to expression (Youngblood et al., 2011). Moreover, IFN-α in association with IRF9 results into the expression of PD-1 by binding to the pdcd1 promoter leading to impairment of T-cells. In case of chronic infections, PD-1 is known to express in T-CD8 cells which are exhausted and is followed by binding of FOXO1 transcription factor to PD-1 promoter for increasing its expression (Xiao et al., 2012). In cancer cells as well, the tumor cell leakage enhances the expression of c-FOS subunit of AP-1, which in turn, enhances the PD-1 expression. However, PD-1 can act in two different ways i.e., it can be harmful and beneficial both (Salmaninejad et al., 2018). It plays a protective role in decreasing the regulation of harmful immune responses or acts as an immunosuppressor and regulates immune tolerance. On the other hand, it can also lead to the dilation of cancer cells by interfering with the protective immune response (Han et al., 2020).

### 2.2 PD-L1 and PD-L2

PD-L1 is a ligand of co-inhibitory receptor PD-1 and is coded by chromosome 9p24.1 located on the CD274gene. It is also called as B7 homolog 1 due to its homology with B7-1, B7-2, and CD274 (Sanmamed and Chen, 2014). Under physiological conditions, constitutive PD-L1 expression takes place in several tissues, mainly in the MSCs, activated T-cells, B-cells, monocytes, DCs, bone marrow derived mast cells and several immune privileged organs (Sharpe et al., 2007). Additionally, PD-L1 expression can be stimulated by γ-chain cytokines and IL-21 in T-cells and CD19^+^B cells respectively. Lipopolysaccharide (LPS) or B-cell receptor activation in B-cells is also known to stimulate PD-L1 expression. IFN-γ in monocytes and non-lymphoid cells like endothelial cells can also stimulate the expression (Ohaegbulam et al., 2015). PD-L1 is extremely conserved evolutionarily which reveals its functional importance. The PD-L1 is often seen at the site of inflammation and on tumor cells of distinctive origin which suggests the wide disposition of PD-L1 in various cellular localizations (Ji et al., 2015). These involve membranous PD-L1 (mPD-L1), nuclear PD-L1 (nPD-L1), serum PD-L1 (sPD-L1), cytoplasmic PD-L1 (cPD-L1) and exosomal PD-L1. It is present as an acquired immune mechanism in cancer cells to skip anti-tumor responses. It is linked with immune surroundings rich in CD8 T-cells, Th1 cytokines, chemokines, and interferons. Studies revealed that IFN-γ results into increased expression of PD-L1 in acute myeloid leukemia via MEK/ERK and MYD88/TRAF6 pathways (Bellucci et al., 2015). IFN-γ activates protein kinase D isoform 2 (PKD2), which is crucial for regulating PD-L1. The inhibition of PKD2 prevents PD-L1 expression and induces an antitumor immune response. NK cells secrete IFN-γ via Janus kinase (JAK) and by activating signal transducer and activator of transcription 1 (STAT1) transcription pathways resulting into enhanced PD-L1 expression (Bellucci et al., 2015). Additionally, PD-L1 expression is regulated by IFN-γ released by T-cells *via* JAK/STAT/IRF1 pathway. PD-L1 is the protein receptor encoded by the CD274 gene, and is located on chromosome 9 in humans. The mRNA expression of PD-L1 takes place by the production of two alternative transcripts of CD274 followed by translation of PD-L1 protein receptor. The longer transcript contains seven exons having a coding sequence of 800 bp and codes for a 33-kDa protein. PD-L1 is a membrane bound glycoprotein with a big extracellular region having immunoglobulin (Ig)-like domains, a small cytoplasmic domain of 30 amino acids and a hydrophobic transmembrane domain (Garcia-Diaz et al., 2017; Khurana et al., 2021). Exon 1 encodes for the 5′-UTR while exon seven codes for the 3′UTR and an intracellular domain. Another transcript is produced through alternative splicing and lack of third exon produces a small 160 amino acid isoform of PD-L1 without having IgV-like domain. Moreover, the promoter region of PD-L1 contains CpG methylation sites alongwith a 220-bp region of epigenetic regulation. The 3′-UTR region of PD-L1 is longer and involves several cis acting elements, which are associated with mRNA decay (Han et al., 2020). Furthermore, PD-L2 is another ligand of PD-1 which shares around 60% sequence homology with PD-L1 in humans (Latchman et al., 2001). As compared to PD-L1, the expression of PD-L2 is limited up to activated DCs, macrophages, bone-marrow derived mast cells and B-cells (Zhong et al., 2007). The expression of PD-L2 could be stimulated by LPS and B-cell receptors in B-cells, and granulocyte-macrophage colony-stimulating factor (GM-CSF), IL-4 on DCs. Both the PD-1 ligands are present in tumor cells and in chronic infection, but the expression of PD-L2 is lower as compared to PD-L1 (Ohigashi et al., 2005).

### 2.3 PD-1/PD-L1-Mediated Signaling

**FIGURE 1:**
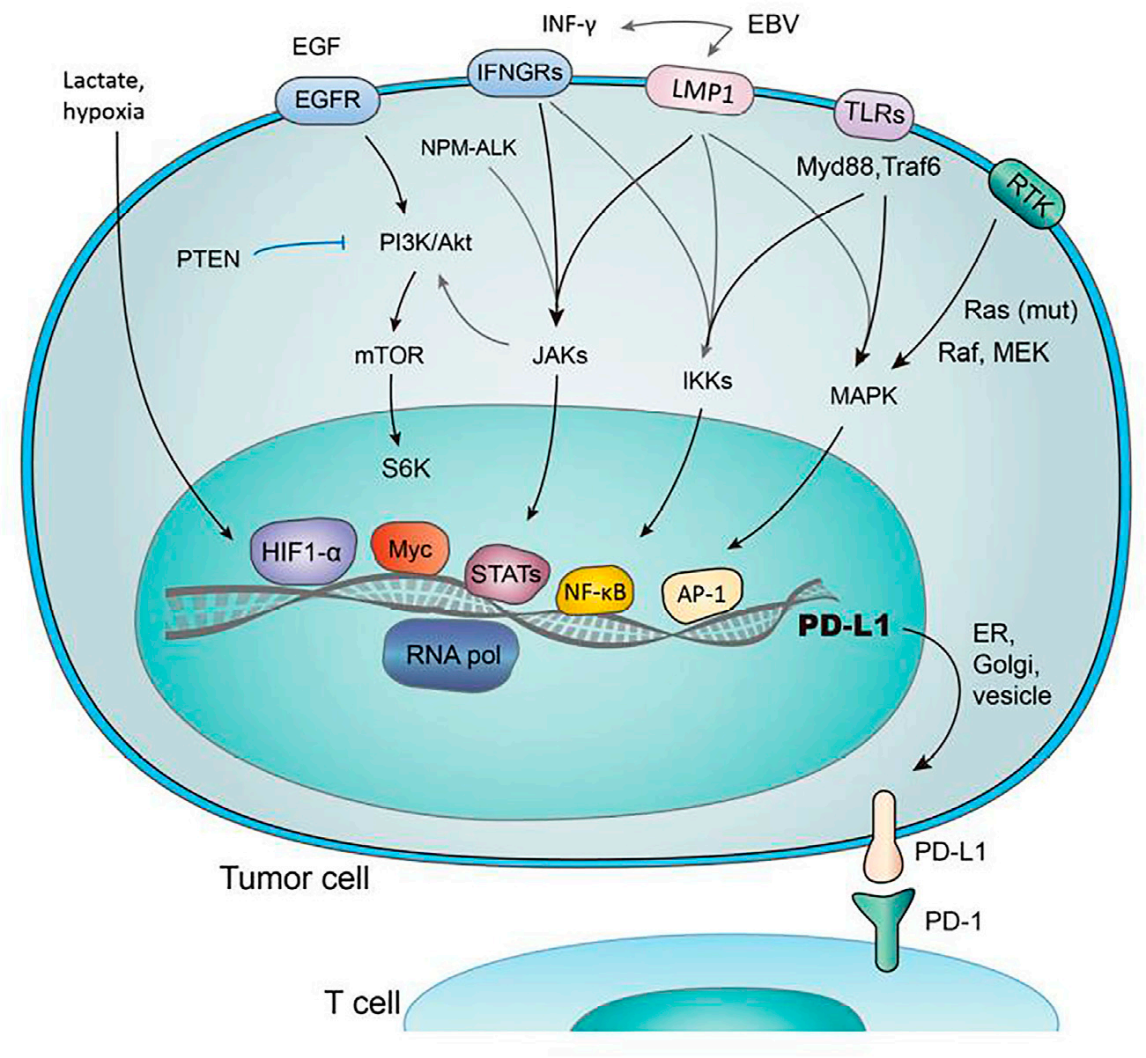
Regulation of PD-1/PD-L1 expression by several pathways. The figure were adapted and reproduced from reference (Wang et al., 2018b) under the Creative Commons Attribution License (CCBY).

The association of PD-1 with its ligands are crucial for regulating a balance among autoimmunity and immune tolerance, alters anti-viral and anti-tumor immune response (Sun et al., 2018). PD-1 transmits signals when it is linked to B-cell receptor (BCR) or TCR and hence, results in the formation of co-inhibitory microclusters with TCR and CD28 (Yearley et al., 2017). It is followed by the inhibition of various processes like preventing cytokine formation, glucose consumption and proliferation of T-lymphocytes. Furthermore, it prevents the expression of transcription factors linked with effector function such as GATA-3, Eomes and T-bet (Nurieva et al., 2006). The ligation of PD-1 results in decreased phosphorylation of protein kinase C, CD3 and ZAP70 (Sheppard et al., 2004). It also prevents the induction of ERK in T-cells and B-cells, and inhibits the phosphorylation of PLC-γ2, Syk, Igβ and calcium mobilization (Freeman et al., 2000). Hence, PD-1 ligation blocks signaling regulated by TCR and hampers the functioning of PI3K-Akt, Hedge-hog, Wnt and Ras/MEK/ERK pathway as shown in Figure 1 (Okazaki et al., 2001).

PD-1 prevents the induction of PI3K-Akt pathway by maintaining the PTEN kinase and phosphatase activity, which results into inhibition of Cdk2 and Skp2 expression as well as an enhancement in p27^kip1^. The binding outcome of PD-1 with PD-L1 varies depending upon the cell type (Parry et al., 2005). In the case of T-cells, PD-1 inhibits its functioning by inhibiting several processes such as disturbing the cell cycle progression and preventing the formation, proliferation and survival of cytokines (Patsoukis et al., 2012). Moreover, PD-1 is linked with TGF-β regulated T_regs_ signaling resulting into their proliferation (Butte et al., 2007). Furthermore, Ras PD-1 controls SWAT3 and gives enhanced effect along with TGF-β. PD-1 ligation also fluctuates the metabolic environment of T-cells and in turn produces an oxidative environment (Tkachev et al., 2015). PD-1 signaling is extensively studied and reviewed in literature. However, our understanding of PD-1 signaling in CLDs is still limited (Dermani et al., 2019). PD-1/PD-L1 blockade based anti-tumor therapy is widely used in various types of cancers. Depending upon the type of cancer, the expression and signaling of PD-1/PD-L1 varies. For instance, in HCC patients, the expression of PD-1 is upregulated in CD8^+^ T cells and an increased number of circulating and tumor-infiltrating PD-1^+^/CD8^+^ T cells are seen which are associated with progression of HCC (Shi et al., 2011; Jubel et al., 2020). In breast cancer, PD-L1 expression is linked to epithelium-to-mesenchymal transition (EMT) in human breast cancer stem cells (BCSCs). It has been reported that the expression of PD-L1 is higher in estrogen receptor (ER)α-negative breast cancer, while a subsequently low expression is seen in ERα-positive breast cancer cell lines. In pancreatic ductal adenocarcinoma (PDAC), enhanced expression of PD-1 was seen in peripheral CD8^+^ T cells, while in bladder cancer it has been reported that autophagy related 7 (ATG7) protein regulates the expression of PD-L1 protein and overexpression of ATG7 results in increased PD-L1 protein levels by stimulating autophagy-dependent degradation of FOXO3A. Additionally, in colorectal cancer (CRC), expression of PD-L1 is generally common in metastatic CRC. PD-L1 expression promotes tumor cells skipping the surveillance of the immune system and increases functioning of T_reg_ in CRC, thus promoting metastasis (Dong et al., 2017; Li et al., 2020).

## 3 Role of PD-1 in Chronic Liver Diseases and Its Relevance as a Therapeutic Target

CLDs can be induced mainly by hepatitis and non-alcoholic fatty liver disease (NAFLD) (Kuol et al., 2018). PD-1 inhibitory pathway aids in regulating T-cell response in acute and chronic liver inflammation and also takes part in expanding inflammation in liver diseases. To understand the functioning of PD-1 and its ligands, their expression profiles are monitored during CLDs in normal and diseased patients (Topalian et al., 2015). It has been observed that patients having CLDs consist of high PD-1 expressing lymphocytes than normal patients. PD-1 ligation on lymphocytes prevent their activation, cytokine production and proliferation (Guan et al., 2017). Normal leukocytes and endothelial cells express a relatively less number of PD-1 ligands while the inducible expression is promoted by inflammatory cytokines like IFN-γ, which can remarkably increase PD-L1 and PD-L2 expression. Chronic liver infection offers various means for modulating the lymphocyte activity through PD-1 ligation (Mandai, 2016).

### 3.1. PD-1/PD-L1 in Hepatocellular Carcinoma

**FIGURE 2:**
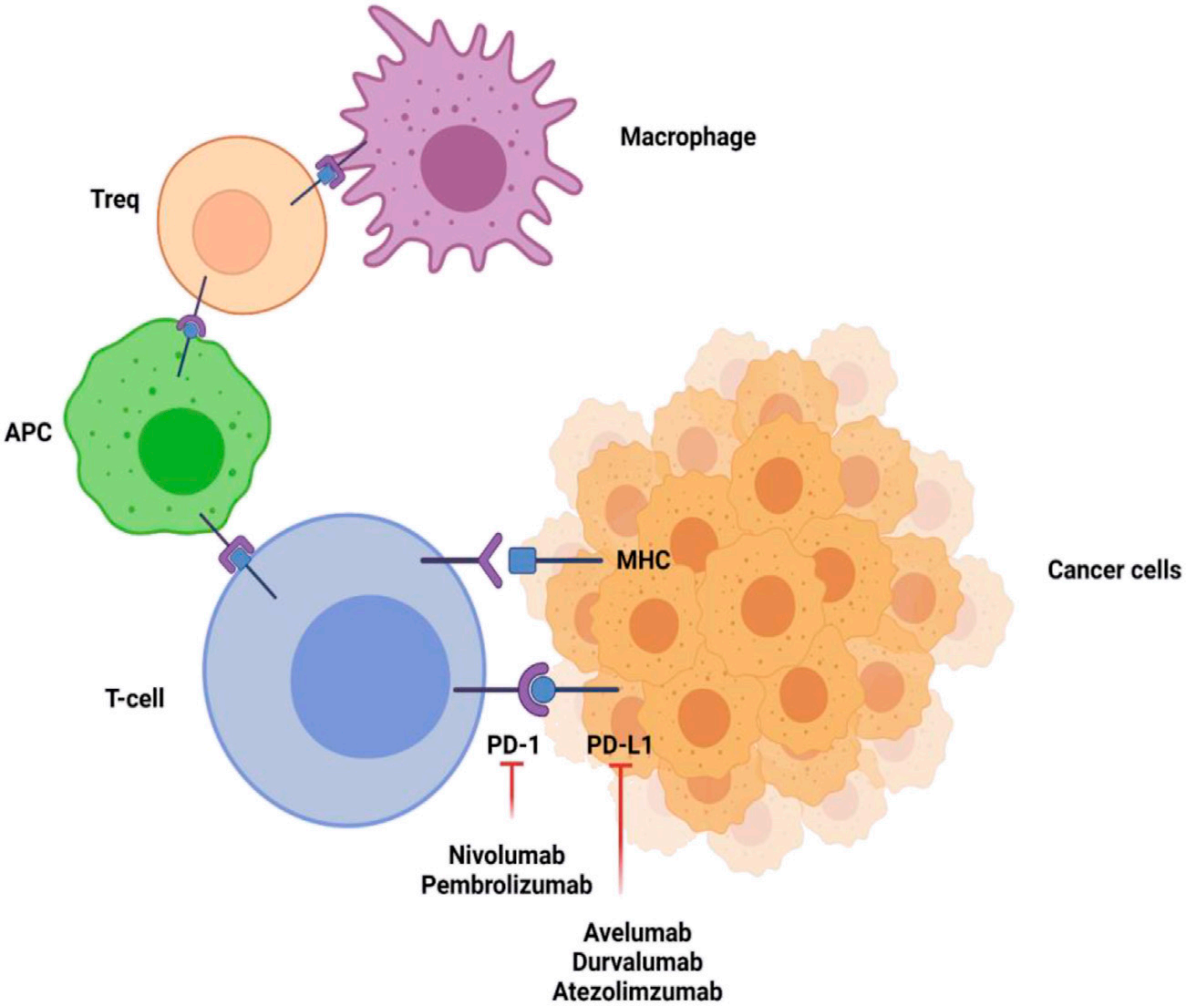
Inhibitory signaling of PD-1 and PD-L1 inhibitors. PD-1 is present on T-cells, antigen presenting cells, macrophages, and regulatory T-cells (T_regs_). Linkage of PD-1 with its ligands results in downregulation of proliferation and immune response of T-cells. Inhibiting the PD-1 or PD-L1 pathway reverses this action and upregulates immune activity. The figure was created with BioRender.com.

Hepatocellular carcinoma (HCC) is considered a very common neoplasia of the liver. It is placed in the second position for causing cancer associated mortality and occupies the 16th position as a common cause of death globally. Based on intensive research in oncology, it is considered that immunotherapy is by far the most effective therapy for HCC. Immunotherapy is effective for HCC as liver is an immune privileged organ in which every immunotherapy based drug has its individual pharmacokinetic profile. Additionally, liver can tolerate to immune response to antigens which is maintained by naive T-cell activation and also by several other immunosuppressive processes. Moreover, HCC is a type of cancer which is linked with inflammation. Immune response takes place by coordination among the stimulatory and inhibitory signals. Among inhibitory signals, PD-1 and PD-1 ligands are considered as most effective and have gained wide attention (Cristescu et al., 2018). In physiological conditions, PD-1/PD-L1 is expressed for regulating self-tolerance and inhibits immune stimulation by T-cell activation. However, cancer cells also show PD-1 resulting into immune escape. Indeed, drugs targeting PD-1 and PD-1 ligands are known to stimulate attentive antitumor effects in HCC as shown in Figure 2. It has become one of the most successful mechanisms in treating HCC in the past few years (Ringelhan et al., 2018). Targeting PD-1 and its ligands is promising due to the possibility of PD-1/PD-L1 staining in HCC patients after surgical resection with prognostic implications (Elsegood et al., 2017). Moreover, serum PD-1 can also be used as a means to monitor prior reoccurrences and to recognize the treatment outcomes. The PD-1/PD-L1 can also be able to serve individual specific treatment for HCC (Wang et al., 2011).

HCC generally takes place due to the occurrence of chronic liver disease (chronic hepatitis infection), metabolic disbalancing or alcoholism. All these promotes exhaustiveness of T-cell consumption and immunosuppressive condition of liver (Shrestha et al., 2018). In tumor growth and progression, immune checkpoints namely PD-1/PD-L1 are highly involved. Studies have shown that HCC patients show high expression of PD-1 in CD8^+^ T-cells and display increased circulating and tumor-infiltrating PD-1^+^/CD8^+^ T-cells, which can be used for disease prediction and to assess postoperative recurrence (Shi et al., 2011). The interaction between activated PD-1 on T-cells and PD-L1 regulates the downstream signaling of T-cell receptor and CD28 co-stimulator signals by adding phosphoryl group to the cytosolic immune receptor tyrosine dependent switch motif resulting into the placement of src homology region 2 domain having phosphatases 1 and 2 (SHP1/2) and slam associated protein (Gao et al., 2009). Additionally, SHP1/2 removes phosphate groups from the TCR and CD28 signaling molecules such as ZAP70 and PI3K, preventing activation of T-cells, increases production of cytokine, promote the expression of pro-apoptotic molecules, and finally lead to apoptosis or anergy of T-cells (Jung et al., 2017). The expression of PD-L1 in cancers headed to exhaustion and unresponsiveness in T-cells, offer immune escapism and progression of tumor. The abrogation of PD-L1 on tumor cells can also increase the sensitivity towards T-cell mediated killing. Intrinsic signaling of PD-L1 is not explored as PD-1, but the treating macrophage with anti-PD-L1 antibodies showed the enhancement in the activity of mTOR pathway and analysis of RNA-seq showed an enhancement in multiple macrophage inflammatory pathways (Kudo, 2019). The immunotherapies focusing on PD-1/PD-L1 showed significant anti-tumor results with very less side effects in patients in advanced stages of cancer. These are reported to enhance the multiplication of tumor-infiltrating lymphocytes and generate a more clonal TCR population in the T-cell population which work against the cancer cells (Nishida and Kudo, 2018).

PD-1 and PD-L1 are largely studied and clinically proven immune checkpoint (Table 1). Studies revealed that PD-L1 prevents tumor cells from direct attack of cytotoxic T-cells. The association of PD-1 present on the cancer cell surface and PD-1 present over CD8^+^T-cells leads to apoptosis and energy in CD8^+^T-cells (Xu-Monette et al., 2017). The expression of the high-mobility group box (TOX) DNA-binding factor in thymocyte selection overlap with the expression of PD-1 and can promote the phenotype and longevity of the exhaustive T-cells. Additionally, PD-L1 can also act as an anti-apoptotic in cancer cells as this may lead to the development of resistance in cancer cells. The inhibitory immune checkpoint expression could be dysregulated in the tumor region, which results into the progression of T-cell mediated immune response via cancer immunotherapy. It has been found that PD-1 pathway downregulates the activation of T-cells in peripheral tissues in the later stages. It suggests that blocking this pathway could be useful in getting possible results. The Food and Drug Administration (FDA) approved anti-PD-1 and anti-PD-L1 antibodies for commercial usage (Gong et al., 2018).

**TABLE 1:** The expression of PD-1 immune checkpoints in liver cancer.

Cancer	No. of tumor samples	Immune checkpoints	Cellular expression	Ref
HCC	217	PD-L1/PD-1	Inflammatory and neoplastic cells	Chen et al. (2017)
HCC	171	PD-1	Inflammatory and neoplastic cells	Wu et al. (2009)
HCC	176	PD-L1	Macrophages	Butte et al. (2008)
HCC	294	PD-L1/PD-1	Tumor infiltrating cells	Butte et al. (2007)
HCC	90	PD-L1	Hepatocytes	Dai et al. (2017)

The intrinsic PD-L1 pathway is abnormally stimulated in many cancers. There are various factors which regulate PD-L1 in cancerous cells such as epigenetic regulation, genetic modifications, oncogenic and tumor suppressor pathways, inflammatory cytokines and various other factors.

### 3.2 Hepatitis

When chronic viral infection takes place, a prolonged persistence of viral antigen is observed. As a result of which there is continuous activation of antigen specific T-cells which induces the entry of antigen specific T-cells into a stage known as T-cell exhaustion (Wang et al., 2011). The impaired T-cells lose various effector activities such as decreased amount of cytokine production specifically IL-2, and decrease in their cytotoxicity and proliferative potential. Moreover, exhaustion of T-cells can lead to increase in the expression of co-inhibitory receptors (Mataki et al., 2007). The expression of PD-1/PD-L1 on antigen specific T-cells and exhaustion of T-cells is studied in various chronic and acute viral infections including hepatitis. Every year around 257 million people are chronically infected with hepatitis B virus (HBV) and an estimate of around 800,000 people die every year because of liver cancer and cirrhosis (Cho et al., 2017). However, the infection with HBV can be prevented via vaccination. Nevertheless, in some cases, the infection is resistant to the treatment. In HBV infection, the PD-1 expression is enhanced in HBV-specific T-cells. The expression of PD-1 is negatively associated with T-cell responses. It has also been studied that PD-1 expression on HBV specific cells in earlier stages of infection is associated with increased serum ALT concentration, hence, pointing that early expression of PD-1 may serve as a biomarker of liver injury (Yao et al., 2007). Recent studies also depicted same outcomes when analysis of PD-1 expression on HBV-specific CD4^+^T-cells were carried out. The blockage of PD-1 pathway is considered as crucial in treating the infectious disease. The outcome and efficiency of PD-1 blocking differ significantly in various studies and among pathogens. In chronic HBV infection, the blocking of PD-1 enhances the proliferation of T-cells and increases the generation of cytokines such as IFN-γ and IL-2 by HBV specific T-cells taken from the liver of patients and peripheral blood region with chronic infection of hepatitis B (Urbani et al., 2008). Tang *et al.*, reported that PD-1 blocking can considerably alter the functioning of CD4^+^T-cell as compared to CD8^+^T-cell function. The PD-1/PD-L1 pathways were earlier reported to downregulate the functioning of HBV specific T-cell function in a transgenic mice model. In this study, the T-cells generated IFN-c which later decreased inspite of the antigen presence in the liver followed by an increment in the expression of PD-1 (Hofmeyer et al., 2011). Blocking of this pathway could delay the downregulation of virus-specific T-cells. Moreover, the occurrence of virus specific T-cells in peripheral blood of patients showed enhanced PD-1 expression and were functionally exhausted while the recovered patient showed lower expression of PD-1. The exhaustion stage of T-cells is reversible i.e., after the blocking of PD-1/PD-L1 pathway, the impaired T-cells are recovered completely (Jin et al., 2010). Similar pattern is observed in case of HCV infection i.e., they also exhibit enhanced expression of PD-1 with decreased effector functions however, by blocking the PD-1/PD-L1 pathway, the situation is restored. Apart from enhanced PD-1 expression, the PD-L1 expression is also increased in hepatic APCs which are responsible for hypo-responsiveness of T-cells in chronic hepatitis infection (Wang et al., 2011). Moreover, along with enhanced PD-1 expression, the dysfunctional HBV/HCV specific CD8^+^ T-cells in the liver exhibit decreased expression of CD28 and CD127, revealing much severe condition (Gehring et al., 2009; Hsu et al., 2010; Li et al., 2020). In contrary, the peripheral dysfunctional T-cell displayed a good expression of CD127, which possibly suggests a lower functional exhaustion (Okazaki and Honjo, 2007). The HBV/HCV infection in liver resulted in exhausted T-cell but the blocking of PD-1/PD-L1 generated different responses in both types of infection. Studies showed that the liver resident HBV specific T-cells are recovered much easily functionally after PD-1/PD-L1 blockade as compared to T-cells which are present at the periphery (Nakamoto et al., 2008). However, no functional restoration of HCV specific T-cell was monitored after PD-1/PD-L1 blockade. These contradictory results signify the specificity of virus specific T-cells in different stages of disease. As discussed earlier, chronic hepatitis viral infection leads to increased PD-1 expression which is linked with dysregulation of T-cells and persistent load of viral antigen thereby favoring chronic hepatitis infection while limiting the immunopathogenesis (Dai et al., 2014). Studies also revealed that chronic HBV infected patients when provided with PD-1 inhibitors, an increment in HBV specific CD8^+^ T-cell is observed (Jin et al., 2010). Immune checkpoint mediated immune restoration can also be associated with severe liver injury and inflammation, liver failure and chances of HCC. The immunosuppressive molecule, PD-1, is associated with the course of HCC and HBV infection. It has been documented that HBV infection stimulates immunosuppression and as the infection progresses, it promotes peripheral immune tolerance which results in oncogenesis, because of impaired surveillance of immune system. T_regs_ plays a major immunosuppressive role as they secrete cytokines namely TGF-β, IL-10, and IL-35, and also inhibit the activity of Th_1_ or Th_2_. It has also been reported that in HBV^+^ HCC, the number of T_regs_ were more as compared to HBV^−^ HCC patients. The increased expression of genes associated with IL-10 pathway, FOXP3, and the immunosuppressive molecules namely cytotoxic T-lymphocyte-associated protein 4 (CTLA-4) and lymphocyte-activation gene 3 (LAG3) were seen in HBV-infected HCC. Additionally, resident memory T cells and myeloid-derived suppressor cells were also enriched in HBV-infected HCC. All these cells promote continuous immune suppressive effects in HBV which finally progresses into the development and progression of HCC. Additionally, the genome of virus is integrated in the DNA of hepatocytes and produces viral proteins. Hepatocytes expressing viral protein can be targeted by nonspecific and uncontrolled immune response. However, the immune checkpoint inhibitor mediated toxicity can be managed by treatment with corticosteroids (Cho et al., 2017).

### 3.3 Acute Liver Injury

PD-1 co-inhibitory receptor aids in immunoregulation by decreasing the initial activation of T-cell, hampers T-cell effector functions and differentiation (Dyck and Mills, 2017). It has been well documented that signaling of PD-1 through T-cells restricts the immune mediated tissue injury during the course of infection. Additionally, PD-1 also restricts the activation of self-reactive T-cells (Sage et al., 2018). The immunoregulation of PD-1during inflammation allows foreign microorganisms to escape the immune defense mechanisms of the host. Increase in the expression of PD-1 is noticed in acute liver infections caused by viruses (i.e., HBV and HCV), and bacterial infections (Sharpe et al., 2007). During acute liver injury, specific T-cells get activated with upregulation of various cell types via pattern recognition receptor signaling or indirectly by allowing the release of inflammatory cytokines. The function of PD-1 in acute infection revealed that PD-1 is associated with proving protection against lethal immunopathology (Dong et al., 2019; Allawadhi et al., 2021). However, PD-1 is not much explored in acute liver injury, and few studies suggest that the expression of PD-1 acts as a crucial biomarker for predicting the outcomes of associated diseases (Erickson et al., 2012). By utilizing the expression of PD-1 as a marker of activation or dysfunction of immune cells in acute injury may offer several benefits when considering the treatment strategies to follow.

## 4 Clinical Development of PD-1/PD-L1 Inhibitors/Antibodies for Liver Diseases

The inhibition of association among PD-1 and PD-L1 results into promising and reliable anti-tumor therapy against several different types of tumors. Drugs or inhibitors targeting PD-1/PD-L1 have the potential to bring revolution in treating HCC. From the past few years, there has been a remarkable progress in the field of oncology and various immune checkpoints inhibitors have been studied with immense potential in the prognosis of cancer (Hamanishi et al., 2016). The PD-1 and PD-L1/PD-L2 inhibitors are recognized as clinically useful inhibitors. Some of the FDA approved drugs against these immune checkpoints are already available in the market (Xu-Monette et al., 2017). Moreover, several trials are in progress for investigating the blockage of immune checkpoints in liver cancer and fortunately, promising early signs have also been reported as enlisted in Table 2.

**TABLE 2:** Clinical trials with PD-1/PD-L1 therapy against liver cancer.

Cancer type	Number	Study arms (combinational)	Stage	Status	Trial NCT
HCC	35	Nivolumab	I	Recruiting	NCT02837029
HCC	154	PDR001	I	Recruiting	NCT02947165
HCC	114	Durvalumab	I	Recruiting	NCT02572687
HCC	51	Durvalumab	I	Recruiting	NCT02740985
HCC	75	Nivolumab	I/II	Recruiting	NCT02423343
HCC	620	Nivolumab	I/II	Recruiting	NCT01658878
HCC	108	PDR001	I/II	Recruiting	NCT02795429
HCC	50	Prembrolizumab	I/II	Recruiting	NCT02886897
HCC	15	Prembrolizumab	I/II	Recruiting	NCT02940496
HCC	50	Nivolumab	I/II	Recruiting	NCT02859324
HCC	90	Durvalumab	I/II	Recruiting	NCT02821754
HCC	620	Nivolumab	I/II	Recruiting	NCT01658878
HCC	28	Pembrolizumab (Keytruda)	II	Recruiting	NCT02658019
HCC	440	Durvalumab	II	Recruiting	NCT02519348
HCC	726	Nivolumab	III	Recruiting	NCT02576509
HCC	408	Prembrolizumab	III	Non-recruiting; Active	NCT02702401
HCC	1,200	Durvalumab	III	Non-recruiting	NCT03298451

Reports suggest that PD-L1 is mainly pro-tumorigenic in a variety of cancers, but functions as a tumor suppressor gene in lung cancer. Similarly, PD-1 also shows differential function in different cancers which needs further investigation (Cottrell and Taube, 2018). Though, presently, there are few reports which show how these immunotherapeutic drugs alter these intrinsic pathways. It has been shown that blocking of PD-1 on CTLs in a mouse model has the capacity to stimulate the PD-L1-NLR family pyrin domain containing 3 (NLRP3) inflammasome signaling pathway which in turn induces the placement of myeloid-derived suppressor cells (MDSCs) and promotes infiltration in the tumor cells (Mahoney et al., 2015). MDSCs have the potential to inhibit T-cell function and thereby, can decrease the immune response and promote development of resistance against anti-PD-1 therapies. Additionally, investigation of the immunotherapeutic drugs namely Atezolizumab was done on breast cancer cells *i.e.*, MDA-MB-231 (Zhao et al., 2020). For monitoring the gene expression after drug administration, RNA-seq was used and it has been revealed that the expression of genes responsible for migration, EMT, metastasis, hypoxia and proliferation were reduced (Wang et al., 2018a). This outcome depicted that Atezolizumab has the potential to alter the gene expression in PD-L1 signaling in cancer cells. Moreover, the anti-PD-L1 antibodies namely Pembrolizumab, Nivolumaband and Atezolizumab on different cell lines were monitored (Jiang et al., 2020). These were reported to upregulate the cell proliferation in comparison to isotype control *in vitro*. Similarly, treatment with monoclonal antibodies for inhibiting PD-1/PD-L1 mediated activation of phosphoinositide 3-kinase (PI3K) and mitogen-activated protein kinase (MAPK) pathways by adding a phosphoryl group to AKT and ERK1/2, respectively, promoting cancer cell proliferation *in vivo* (Zheng and Zhou, 2015). These studies demonstrate that immunotherapeutic antibodies have the potential to alter the functioning of PD-L1/PD-1 and may induce other pathways through which tumor cells generate resistance against targeted PD-1/PD-L1 therapy (Hamanishi et al., 2016).

### 4.1 Inhibitors of PD-1/PD-L1

The inhibitors targeting PD-1/PD-L1 have been known to play considerable role in cancers. Herein, we have discussed some of the important inhibitors with potent activity.

#### 4.1.1 Nivolumab

It is available as human monoclonal antibody which prevents the association of PD-1 with its ligand PD-L1. Treatment of advanced liver cancer with Nivolumab resulted in partial remission (Han et al., 2020). Increase in aminotransferases was observed during treatment which suggests that drug administration in combination should be given after risk monitoring. It has been approved for patients who failed to respond to platinum based immunotherapy. However, immunotherapy after few chemotherapy sessions worked with durable treatment benefits (Zhao et al., 2019).

#### 4.1.2 Pembrolizumab

It is one of the approved anti-PD-1 inhibitors and this immunotherapeutic inhibitor has shown improved tumor response (Joseph et al., 2018). For instance, pembrolizumab has been studied in phase Ib KEYNOTE-012 and single-arm phase-II KEYNOTE-055 trials and it showed 18% response rate and an average survival of 6–8 months in treated, recurrent, and metastatic patients. Moreover, the combinational therapy *i.e.,* administration of Pembrolizumab with Trastuzumab has been utilized in cancer patients with PD-L1-positive and resistant to other treatments (Pelster et al., 2020).

#### 4.1.3 JQ1

The therapeutic effects of JQ1 have been monitored at gene level i.e., the expression of PD-L1 mRNA and protein in different cancer cell lines including the liver cell line. It was reported that JQ1 works in a dose dependent manner and can prevent cell proliferation in a dose dependent manner. PD-1 is downregulated in the primary culture of liver cancer cell lines when treated with JQ1 (Liu et al., 2019). Moreover, reduction in PD-L2 expression has been seen in cells treated with JQ1 which revealed that JQ1 regulates PD-1/PD-L1 pathways (Han et al., 2020).

#### 4.1.4 Atezolizumab

It is a human anti-PD-L1 monoclonal antibody which functions by preventing the association of PD-1 with its ligands i.e., PD-L1/PD-L2, and in turn promotes T-cell mediated immunity. It has been administered in combination with Bevacizumab (an anti-VEGF antibody) and the efficacy was compared with Sunitinib. Results indicated that Atezolizumab/Bevacizumab combination showed improved outcomes with ≥1% PD-L1 expression of tumor-infiltrating immune cells when monitored *via* immunohistochemistry (Crist and Balar, 2017).

#### 4.1.5 Avelumab

It is a PD-L1 inhibitor human IgG1 monoclonal antibody. The administration of this inhibitor in phase 1b trial in metastatic or recurrent cancer patients showed that the expression of PD-L1 was downregulated in immune and tumor cells. It has also been studied that a combination of Avelumab and Axitinib can result in a positive response in 58% of the patients and the rate of cancer relapse was controlled upto 78% in a group study of 55 patients (Han et al., 2020).

#### 4.1.6 Cemiplimab

Cemiplimab functions by binding to PD-1 and prevents its association with its ligands and acts as human PD-1 monoclonal antibody (Han et al., 2020). Phase I studies suggests that various human IgG4 monoclonal antibodies functions by inhibiting PD-1 and PD-L2 in later stages of cancer and resulted into the FDA approval of first PD-1 inhibitors namely Pembrolizumab and Nivolumab. Additionally, inhibitors of the immune checkpoints have also been approved for the treatment of malignant cancer. After the approval of first immune checkpoint inhibitor i.e., Pembrolizumab for treating advanced cancers, which opened the doors for developing potent immune checkpoint inhibitors. Currently, there are many FDA approved PD-1 and PD-L1 inhibitors used for the therapy of nine different types of cancer (Macek Jilkova et al., 2019). We have enlisted the PD-1/PD-L1 inhibitors in Table 3 and Table 4. Additionally we have enlisted different cells expressing PD-1 and its ligands and their signaling mediators in Table 5.

**TABLE 3:** Pre-clinical studies with PD-1 immune checkpoints.

Cancer	Number	Immune checkpoints	Treatment	Target	Ref
HCC	71	PD-L1 and PD-1	PD-L1 Ab and PD-1 Ab	CD8^+^T-cells and Kupffer cells	Li et al. (2016)
HCC	not reported	PD-L1	PD-L1 shRNA	HCC cell lines	Lienlaf et al. (2016)
HCC	59	PD-1	PD-1 antibodies	tumor-infiltrating T-cells	Chang et al. (2017)

**TABLE 4:** List of clinically approved PD-1/PD-L1 inhibitors.

Agent	Target	Names	Antibody clone	Company
Pembrolizumab	PD-1	KEYNOTE	22C3 (Dako)	Merck
Atezolizumab	PD-L1	IMVigor, POPLAR, OAK	SP142 (Ventana)	Genentech
Nivolumab	PD-1	CheckMate	28-8 (Dako)	BMS
Avelumab	PD-L1	JAVELIN	73-10 (Dako)	Pfizer, Merck
Durvalumab	PD-L1	Study 1,108	SP263 (Ventana)	AstraZeneca

**TABLE 5:** Cells expressing PD-1 and its ligands via different signaling molecules.

Immune checkpoint protein/ligands	Cells	Signaling/stimulatory molecule involved	Pathway	Ref
PD-1	T cells (CD4, CD8, T_regs_)	IL-10 and TGF-β, NOTCH, Forkhead box protein (FOXO1), interferon regulatory factor (IRFs) and nuclear factor of activated T-cells (NFAT)	Phosphoinositide 3-kinase (PI3K)/AKT, phospholipase C-γ (PLCγ), ERK, JAK/STAT/IRF1	Dong et al. (2017); Cagle et al. (2018); Sordillo et al. (2021); Touboul et al. (2021)
	Activated B cells			
	NK cells			
	Macrophages			
	Dendritic cells			
	Langerhans cells			
PD-L1	T cells (CD4, CD8, T_regs_)	IFN-γ, Lipopolysaccharide (LPS), B-cell receptors in B-cells (BCR)	JAK/STAT/IRF1, MEK/ERK and MYD88/TRAF6	Bardhan et al. (2016); Tewari et al. (2017); Lu et al. (2019)
	Activated B cells			
	Macrophages			
	Mesenchymal stem cells (MSCs)			
PD-L2	Dendritic cells	GM-CSF, IL-4	Phosphoinositide 3-kinase (PI3K)/AKT	Xing et al. (2018); Lu et al. (2019)
	Mast cells			
	Macrophages			

## 5 Toxicological Issues of PD-1/PD-L1 Inhibitors/Antibodies

Generally, there are very limited reports of toxicities caused by anti-PD-1/PD-L1 monoclonal antibodies. Further, the toxicities that exist are generally not very severe. In the following section, we have discussed some of the toxicological issues of anti-PD-1/PD-L1 inhibitors in patients administering as a single agent or in combination with other standard agents such as targeted therapy, chemotherapy and other immunotherapeutic drugs (Naidoo et al., 2015). Some of the commonly associated toxicities of anti-PD-1/PD-L1 therapy are discussed below.

### 5.1 Fatigue

Fatigue is considered the most common side effect of anti-PD-1/PD-L1 agents. It has been revealed that Nivolumab administration resulted in fatigue in 16–24% of patients. Additionally, it has been observed that anti-PD-1 agents showed 16–37% chances of fatigue while anti-PD-L1 agents showed 12–24% incidence (Brahmer et al., 2015). However, the incidence of fatigue was increased and range from 21 to 71% when anti-PD-1/PD-L1 agents were administered with other chemotherapeutic drugs, anti-angiogenic agents and targeted therapies. Fatigue associated with combinational therapy can also generate other systemic manifestations like illness and cytokine release (Bendell et al., 2015). The mechanism of associated fatigue is unknown, however, it is suggested that it is not dose dependent (Weber et al., 2015a).

### 5.2 Fever and Chills

Fever and chills are generally associated with other immunotherapeutic agents also including vaccines, immune modulating antibodies or targeted therapies. The basic mechanism behind these toxicities is the release of cytokines and non-specific stimulation of the immune system and related response. However, these are managed easily by providing antipyretics and non-steroidal anti-inflammatory drugs (Weber et al., 2015a).

### 5.3 Organ Specific Toxicities

#### 5.3.1 Dermatological Toxicity

The most commonly immune checkpoint monoclonal antibody associated toxicity is skin rash and it takes place after the second phase of clinical practice. The skin rash can be of any type such as maculopapular, urticarial dermatitis, follicular, papulopustular, or Sweet’s syndrome. It has been observed that administration of Pembrolizumab and Nivolumab resulted in the development of rashes in 39 and 34% of patients, respectively (Postow, 2015). A study revealed that the combination of Pembrolizumab with Ipilimumab developed more chances of vitiligo in patients i.e., in 10% while only 2% when administered with single Ipilimumab (Weber et al., 2015b; Robert et al., 2015). The dermatological toxicity may be due to the blockage of some common antigens which is co-expressed in tumor cells of patients and on dermal-epidermal junction or any other layer of the skin. Other mucosal associated toxicities are oral mucositis, sicca syndrome and gingivitis. However, these can be managed with symptomatic medication. These dermal toxicities can be easily managed by prescribing corticosteroids or antipruritic drugs such as antihistamines, NK-1 receptor inhibitors and GABA agonists (Postow, 2015). However, before the initiation of the treatment, dermatological examination should be followed which involves standard clinical evaluation and monitoring the serum levels. Histological examination is also recommended for dermatitis. It has been revealed that in less than 5% cases, discontinuation of treatment occurs with dermatological toxicity.

#### 5.3.2 Endocrine Toxicities

Immune checkpoint inhibitors can also affect the functioning of endocrine glands. The evaluation of endocrine dysfunction is critical as they are non-specifically associated with other symptoms like headache and fatigue (Ryder et al., 2014). Before the clinical administration of drugs, hypophysitis should be evaluated by evaluation of the levels of prolactin, T4, TSH, LH, FSH, ACTH, and cortisol and pituitary inflammation. The chances of hypophysitis are only 1–6% with alone anti-PD-1/PD-L1 monoclonal antibody and a higher incidence of 2–10% is observed in some of the combinational therapies. The endocrinal toxicity is thought to be developed due to the generation of humoral immune response against pituitary along with the association of compliment system (Postow, 2015).

#### 5.3.3 Hepatic Toxicity

Hepatic toxicities associated with blocking of immune checkpoints involve an asymptomatic increase in aspartate transaminase (AST) and alanine aminotransferase levels (ALT) (Postow, 2015). The increased elevation in AST/ALT ratio has been shown in HCC with∼20% elevation with anti-PD-1 inhibitors. Ipilimumab has been reported to be linked with immune checkpoint induced hepatitis therefore, it is not used in hepatitis induced injury (El-Khoueiry et al., 2015).

#### 5.3.4 Pneumonia

It is generally the inflammation in the parenchyma of lungs and has been documented with anti-PD-1/PD-L1 inhibitors in 10% of the treated patients singly or in combination with other drugs. Though the incidence of pneumonia related toxicities increases when combinational anti-PD-1/PD-L1 monoclonal antibodies are administered (Postow, 2015). The arousal of pneumonitis symptoms varies considerably from 7.4 to 24.3 months after starting the therapy. Patients are known to experience cough, pain, fever, chills and shortness of breath. However, this toxicity can be managed by rational evaluation and giving corticosteroids and other immune suppressors such as infliximab or cyclophosphamide (Naidoo et al., 2015).

### 5.4 Rare Toxicities

#### 5.4.1 Neurological Toxicities

It has been reported that the combination of anti-PD-1/PD-L1 monoclonal antibody with anti-CTLA-4 monoclonal antibody may result in the incidence of myasthenia gravis. The administration of Ipilimumab singly is linked with several neurological syndromes like transverse myelitis, aseptic meningitis, enteric neuropathy and Guillain-Barre syndrome. This toxicity can be managed by corticosteroids and other neurological evaluations (Postow, 2015).

#### 5.4.2 Ocular Toxicity

Studies revealed that the administration of anti-PD-1 therapy either alone or along with other therapies can develop uveitis (Robert et al., 2015). Additionally, the single use of Ipilimumab has been reported to develop uveitis. However, it can be managed by oral or topical corticosteroid solution in consultation with a specialist (Postow, 2015).

#### 5.4.3 Renal Toxicity

The administration of anti-PD-1 therapy has also been reported to cause intestinal nephritis. Moreover, the combinational therapy of Nivolumab and Ipilimumab can also cause intestinal nephritis (Hamid et al., 2013). The symptoms are not clearly visible as a result of toxicity caused by anti-PD-1/PD-L1 monoclonal antibody. The reports of renal failure have also been seen when Nivolumab is given in combination with platinum mediated chemotherapy. However, the use of corticosteroids is usually helpful in improving the situation (Wolchok et al., 2013).

#### 5.4.4 Pancreatic Toxicity

The use of anti-PD-1/PD-L1 monoclonal antibody can cause upregulation in the lipase levels. However, the cases of pancreatitis are very low and the clinical evaluation of pancreatitis requires regular assessment of lipase and amylase. Routine evaluation of these enzymes should be followed in asymptomatic patients also (El-Khoueiry et al., 2015).

## 6 Conclusion

Conclusively, PD-1/PD-L1 plays a crucial role in various liver diseases including HCC, making it a potential therapeutic strategy for the treatment of CLDs. Immunotherapy is considered as a revolutionizing therapy providing survival benefits to a large number of patients. A large number of hepatocellular cancer cells and hepatitis infected cells exhibit increased expression of PD-1/PD-L1, which makes PD-1/PD-L1-targeted inhibitors as the promising treatment strategy. Immunotherapy based on PD-1/PD-L1 inhibitors has the potential to become the next logical step in the treatment of CLDs. However, along with the opportunities, there are various challenges associated with PD-1/PD-L1 blockade therapy such as dosage standardization, safety measures, half-life, and of course efficacy. Further studies are required which may aid in overcoming the challenges associated with PD-1/PD-L1 inhibitors and to promote their wider therapeutic applications.
